# Nevogenesis: A Benign Metastatic Process?

**DOI:** 10.5402/2011/813513

**Published:** 2011-04-05

**Authors:** Andrew L. Ross, Margaret I. Sanchez, James M. Grichnik

**Affiliations:** ^1^Department of Dermatology and Cutaneous Surgery, Miller School of Medicine, University of Miami, Miami, FL 33136, USA; ^2^Melanoma Program, Sylvester Comprehensive Cancer Center, University of Miami, Miami, FL 33136, USA; ^3^Interdisciplinary Stem Cell Institute, Miller School of Medicine, University of Miami, Miami, FL 33136, USA

## Abstract

It is generally accepted that cutaneous nevogenesis is a localized event that occurs exclusively in the dermis and/or epidermis. However, the discovery of nevocytes circulating in the peripheral blood suggests that other, more systemic, benign metastatic processes could also be involved. The theoretical role of lymphatic and hematogenous dissemination of loosely adherent, immature nevus progenitor cells in the development of nodal nevi and eruptive melanocytic nevi will be reviewed.

## 1. Introduction

The process of nevogenesis is generally thought to be the result of a localized dermal and/or epidermal event. In the localized model, a single nevus progenitor cell located in the skin undergoes a transforming event that leads to local migration and proliferation creating a single nevus in the vicinity of where the progenitor cell was transformed. 

Though more controversial, it is also possible that nevogenesis occurs through a more systemic, benign metastatic process. In the systemic model, a single transformed immature nevus progenitor cell gives rise to multiple systemic nevi through lymphatic and hematogenous dissemination. This model provides an alternative explanation for the development of both simultaneous eruptive nevi and nodal nevi.

## 2. Model for Benign Metastasis

**Figure 1:**
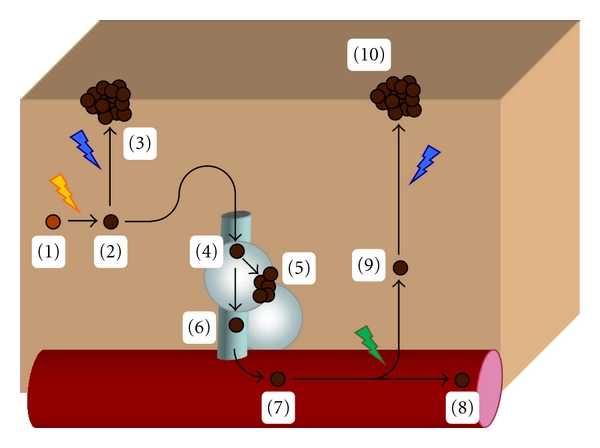
Model of benign metastasis. In this model, a melanocytic stem cell (1) undergoes an initiating event that transforms it into an immature nevus progenitor cell (2). This cell (2) may remain quiescent until local environmental factors stimulate it to proliferate into a nevus (3). Alternatively, this loosely adherent cell could enter the lymphatic system (4). Upon encountering a lymph node, the progenitor cell could either implant in the node and proliferate into a nodal nevus (5) or pass through without being sequestered (6) to eventually reach the circulatory system (7). The progenitor cell would continue to circulate (8) until a transforming event, like a mutation, or environmental conditions signals the cell to implant in the skin (9). The implanted nevus progenitor cell would remain quiescent in the skin until local environmental factors stimulate it to proliferate into a nevus (10).

In the systemic model, a melanocytic stem cell residing in the dermis, or possibly the epidermis, undergoes an initiating event that primes the cell to proliferate excessively (see Figure 1). This nevus progenitor cell would remain quiescent in the dermis until environmental conditions prompted the cell to either (1) undergo localized proliferation to form a nevus at that site, or (2) enter into systemic circulation through a lymphatic or hematogenous route. Lymphatic entry seems most plausible because the loosely adherent nature of melanocytic stem cells may predispose them to be swept into lymphatic channels in the dermis [1]. The initiating event that transforms a melanocytic precursor cell into a nevus progenitor cell may facilitate this process (i.e., inflammation after a sunburn). Direct hematogenous entry is less likely given that it does not readily explain the presence of nodal nevi.

Upon entering the lymph node, the nevus progenitor cell could follow one of two paths. First, it could implant in the node. Upon implantation, the cell could either remain quiescent or migrate into the capsule where it would proliferate into a nevus. Second, its loosely adherent nature could allow it to pass through without being sequestered by the node [2]. The nevus progenitor cells that failed to implant in the node would continue on to enter into the circulatory system. At some point in this process, the nevus progenitor cell would begin to undergo limited division. This could take place in the tissue where the initiating event occurred, within the lymph node, or upon entry into the circulation. The resultant cells would continue to circulate for an indeterminate period of time.

In the course of their travel, the circulating cells would eventually be exposed to a microenvironment that encourages diapedesis and implantation. Depending on the signaling molecules present in the extracellular milieu, the implanted cells could immediately begin to proliferate or remain quiescent until recruited by a change in local signaling molecules.

## 3. Supporting Evidence

One of the most important facts that supports this theory is the existence of nodal nevi. Benign nevocyte aggregates in lymph nodes were first described by Stewart and Copeland in 1931. Since then, their existence has been confirmed in multiple reports [3–9]. Although the existence of nodal nevi could be explained by arrested melanocyte precursor migration during embryogenesis, Patterson has put forth a compelling argument in favor of mechanical transport [10]. It has been suggested that mechanical transport is initiated when a melanoma arises in a preformed nevus and displaces benign nevocytes into the lymphatic system [6, 10]. This hypothesis is supported by the fact that benign nevocytes reported in the literature frequently colocalize with malignant melanocytes in lymph nodes [6]. However, this observation is likely secondary to selection bias, as few healthy individuals commonly undergo lymph node sampling. Additionally, nodal nevi have been described in individuals with other malignancies and individuals with no comorbidities [3, 4]. If nodal nevi are derived from nevocytes displaced from preformed nevi, then it would be expected that all corresponding dermatomes drained by lymph nodes containing nevocyte aggregates should possess a parent nevus. Although Holt et al. did demonstrate that 6 of 8 nodal nevi had an associated cutaneous nevus, this was not always the case [8]. The two remaining cases presented in their report could be accounted for by the systemic model in which cells may pass from tissue and through nodes without requiring the development of a nevus at the cutaneous site.

Another important finding that supports the aforementioned theory is that benign nevocytes have been isolated in the peripheral blood [11]. Direct hematogenous invasion by nevocytes is unlikely given the cells' benign nature. As such, it is probable that hematogenously disseminated nevocytes entered into systemic circulation through the lymphatic system. This implies that loosely adherent nevus progenitor cells collected by lymphatic channels in the dermis are able to traverse the lymphatic chain without being sequestered in the lymph nodes. Consequently, there is evidence to support the notion that upon entering a lymph node, nevus progenitor cells can either implant in or pass through the node to enter the peripheral blood stream.

There is currently no direct evidence that supports the hypothesis that benign nevus progenitor cells in the peripheral blood stream are able to exit the circulation and implant in the skin. However, the phenomenon of epidermotropic metastatic melanoma does suggest that circulating melanocytes, albeit malignant ones, can demonstrate a propensity to implant in either the dermis and/or epidermis [12]. Given the fact that malignant melanoma and benign nevi possess similar growth promoting mutations, it is not unreasonable to propose that they also share the ability to hematogenously disseminate and implant in the skin. This shared ability to metastasize does not necessarily imply that these two very different cells will behave similarly upon implantation. It is likely that additional mutations in senescence pathways permit malignant melanocytes to undergo uncontrolled proliferation [13], while nevus progenitor cells with intact senescence pathways eventually undergo growth arrest. 

The phenomenon of eruptive nevi is characterized by the sudden systemic development of multiple nevi over a short period of time. This clinical scenario has been associated with immune suppression [14–17], pregnancy [18], inflammatory conditions [19], and cytokines [20]. It thus appears that the systemic eruption of nevi is triggered by a discrete systemic event that causes transformed, quiescent nevus progenitor cells located throughout the skin to proliferate. The existence of this susceptible nevus progenitor cell is supported by the fact that not all individuals exposed to these stimuli develop eruptive nevi and that patients who do undergo this phenomenon develop discrete lesions as opposed to general hyperpigmentation. It remains plausible that this systemic population of susceptible nevus progenitor cells was derived from a single cell. Supporting evidence includes a recent case report by Sekulic et al. that demonstrated a BRAF V600E mutation in 17 of 20 eruptive nevi removed from a single patient who was being treated with 6-Mercaptopurine [21]. Although the authors interpreted their results to imply that treatment with this immunosuppressive agent increases mutational frequency, it is also possible that most of these nevi share a monoclonal origin. In the latter scenario, the mutation would have occurred in a progenitor cell whose progeny disseminated systemically.

In summary, given the phenomena of epidermotrophic metastatic melanoma in which tumor cells can focally proliferate in the dermis and migrate into epidermis, eruptive nevi, and the recent finding of circulting nevus cells, it is rational to consider the possibility that nevogenesis occurs by a benign metastatic process.

## 4. Counterpoint

The benign metastasis model is unproven and there exist a number of questions that remain to be answered. One of these questions is if benign nevus progenitor cells do metastasize, then why are nevi not frequently found in other internal organs like the lungs and brain? While it could be argued that these organs do not support implantation and growth of circulating benign nevocytes, this is difficult to reconcile with malignant melanocytes' proclivity for these tissues. Nevertheless, it remains possible that benign melanocytes possess intrinsic characteristics that prevent them from implanting or growing in these tissues. If this is true, the recognition of these characteristics and the discovery of their control mechanisms could have potential therapeutic benefits.

## 5. Conclusion

The systemic dissemination of nevus progenitor cells through lymphatic and hematogenous routes could play a role in nevogenesis.
